# Performance Evaluation of a Nylon-like Polyester Tire Cord Combining the Characteristics of Nylon and Polyester

**DOI:** 10.3390/polym16121645

**Published:** 2024-06-11

**Authors:** Liyong Tian, Yangfan Zhang, Haibin Zhu, Feng Gan, Ningbo Yi, Yancheng Wu

**Affiliations:** 1 College of Textile Science and Engineering, Wuyi University, Jiangmen 529020, China; tly@wyu.edu.cn (L.T.); zyf@wyu.edu.cn (Y.Z.); 2 Indorama Ventures Mobility (Kaiping) Co., Ltd., Kaiping 529300, China; simon.zhu@cn.indorama.net

**Keywords:** nylon-like polyester tire cord, tire performance, indoor testing, rolling resistance, road testing

## Abstract

A nylon-like polyester tire cord, which combined the characteristics of nylon and polyester tire cords, was designed as the carcass reinforcement material used to meet the increasing demands of the tire industry for performance and impact on the environment. Tires’ carcass construction plays a crucial role in affecting handling performance and ride comfort. Small changes in the carcass component can lead to significant improvements in the total tire/vehicle performance. This study evaluated the performance of nylon-like polyester and nylon 6 motorcycle tires. The results showed that the nylon-like polyester tire passed all indoor tests, and post-cure inflation (PCI) could be eliminated, resulting in energy and cost savings. The rolling resistance coefficient of the nylon-like polyester tire was reduced by 6.8% compared to that of the nylon 6 control tire, which could save fuel and have a positive impact on the environment. Nylon-like polyester tire cord extracted from the experimental tire possessed a higher modulus compared to that of nylon 6 tire cord, which could lead to better handling and ride comfort performance. Morphological pictures showed that both nylon-like polyester and nylon 6 cords extracted from tires had a good rubber coverage and comparable adhesion properties.

## 1. Introduction

A tire is considered to be one of the most crucial components in a vehicle system, supporting the vehicle weight, providing a cushioning effect to surface irregularities and providing traction and steering control [1]. The tire, a textile-fiber-reinforced rubber composite, is much more complicated than it appears. Tire cords, as the structural skeleton component, carry most of the load of a tire and maintain its shape [2,3,4]. The overall performance of a tire is highly dependent on the properties of tire cords. As a result, tire cords play a very significant role in a tire’s performance [4]. There are five major synthetic tire cords as carcass material available in the market. These are made from aramid, nylon, polyester (PET), regenerated cellulose (rayon) and polyethylene naphthalate (PEN) fibers [3,4,5,6,7]. Each type of tire cord has its distinct characteristics. Thus, it is a dilemma to choose a single one as the tire reinforcement material. Hybrid cords, however, have added a new element to this dilemma. Hybrid cords [3,5,8], consisting of at least two different fibers, combine the positive characteristics of their components and deliver a better performance to the tire in terms of reducing weight, decreasing rolling resistance or improving dimensional stability. PET and nylon tire cords are currently two of the most popular types of man-made carcass tire cords [7]. In order to synergize the positive properties of both cords, a PET/nylon hybrid cord was designed by twisting nylon and PET fiber filaments together [5,7]. In our previous studies [3,8], a nylon-like polyester tire cord was designed by optimizing the manufacturing process parameters, which combined the positive characteristics of polyester cord with a high modulus and nylon cord with high impact absorbance. The characteristics of this nylon-like polyester tire cord were similar to those of the PET/nylon hybrid tire cord described in other studies [5,7], and yet this newly designed nylon-like polyester cord was composed of a single polyester fiber and simultaneously had a homogeneous surface with a rubber compound matrix.

The properties of this nylon-like polyester tire cord were characterized in our previous studies, including its mechanical and adhesion properties [3,8]. The results showed that the nylon-like polyester tire cords had practicable dimensional stability, a high modulus, comparable adhesion and a favorable fatigue resistance. These characteristics mean that they could provide an alternative to nylon tire cords in motorcycle tires. In this study, motorcycle bias tire experiments were carried out to evaluate the performance of nylon-like tire cord. There are fewer motorcycle tires using standard polyester tire cord as the carcass material due to its poor elongation and shock absorbance; instead, nylon 6 is coming to be the most used in motorcycle tires. Thus, nylon 6 tire cord was adopted as a reference in this study to evaluate the performance of the nylon-like polyester tire cord.

## 2. Materials and Methods

### 2.1. Materials

This nylon-like polyester tire cord (fabric) and standard high-modulus low-shrinkage (HMLS) polyester tire cord were prepared at the Performance Fibers (Kaiping) Company Limited. Reference tire cords used in this paper, PA6 and PA6.6 tire cords, were procured from Shenma Industrial Co., Ltd., Pingdingshan, China. Experimental motorcycle tires with different tire cord fabrics were comparably produced by a designated tire manufacturer. The densities of the tire cord fabrics nylon-like polyester and nylon 6 were 23 and 20 EPI (ends per inch), respectively. The nominal size of a 3.00–18 two-ply motorcycle bias tire (6PR 50 L tube type) was used in this study to evaluate tire performance levels.

### 2.2. Methods

**Figure 1:**
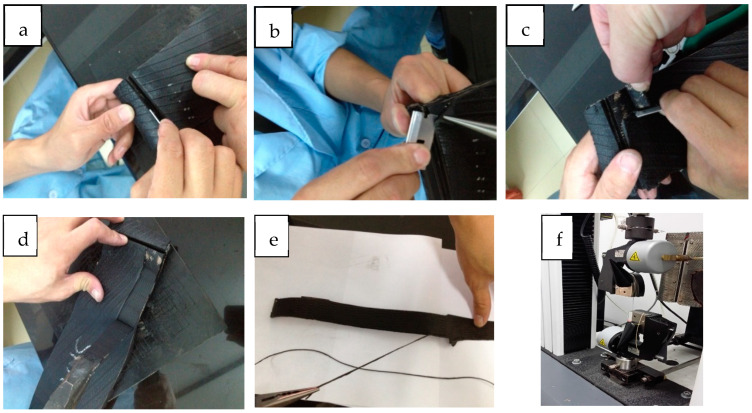
Method of extracting tire cords from motorcycle tires.

Extracting tire cords from motorcycle tires involves the following steps (Figure 1): (a) Cutting off a 30 cm length section of the tire along the circumference direction and removing the steel bead rings on both sides with a single-sided blade. (b and c) Separating a thin (15 mm) piece of carcass ply with a single-sided blade, from the motorcycle tire’s 2-ply carcass with a bias structure. (d) Using a pair of pliers to peel off the thin piece of carcass ply with a steady pull, minimizing extra damage to the tire cords. (e) Separating each tire cord of the carcass strip at one end, and then peeling off each cord with a pair of pliers steadily. (f) Trimming any rubber coverage of the extracted single tire cords carefully without causing any damage, to measure its mechanical properties. Extra rubber cover can strengthen a tire cord’s breaking strength; in order to minimize the effect of rubber coverage, the extra rubber cover needed to be trimmed carefully with a pair of scissors. In this case, due to the motorcycle tire’s bias structure, the length of the extracted tire cord was very short; thus, the working length in the Instron tensile tester was adjusted to 190 mm.

### 2.3. Characterizations

#### 2.3.1. Mechanical Measurement

The normal tire cord tensile measurements were taken, according to ASTM D885, in an Instron tensile tester 3367 at room temperature (25 °C), with the cross head moving at a speed of 254 mm/min and a gauge length of 254 mm. The toughness and other mechanical data were obtained automatically using Instron Bluehill Universal 4.

Tire cords extracted from motorcycle tires were evaluated in the same way but with a gauge length of 190 mm.

Each set of mechanical properties was determined with the same set of testing parameters so the data were reliable and comparable. The final values from this test were averages of 10 test runs.

#### 2.3.2. Thermal Shrinkage Measurement

A thermal shrinkage test of tire cord was carried out, according to ASTM D4974, on a Testrite tester. The test conditions were 177 °C for 2 min with a pre-load of 0.05 g/D. The values obtained in this test were averages of 3 experimental runs.

#### 2.3.3. Indoor Testing of Tires (Endurance, High Speed, Plunger Energy and Dimensions)

Motorcycle experimental tires were mounted on the test rims and then placed on the testing instruments to evaluate the following properties: endurance, high speed, plunger energy and dimensions after inflation. These tests were carried out according to the IS 15627 2005 protocol [9]. The values compiled in these tests were averages of 3 experimental runs.

#### 2.3.4. Tire Dimensional Stability

The increment of OD (overall diameter) and SW (section width) over time was adopted to evaluate the tire dimensional stability. Experimental tires were mounted on the test rims and inflated to a maximum air pressure of 280 kPa at room temperature under the IS 15627 2005 protocol [9]. Then, the circumference of a tire was measured using a soft ruler, and the section widths were determined at 4 different quartering points with a Vernier caliper; the final value of this test was the average of those 4 points.

#### 2.3.5. Rolling Resistance

A rolling resistance test was carried out, according to the ISO 28580 2009 protocol [10] at the Smithers Rapra Suzhou laboratory. A 1.707 m drum-80 Grit 3 mite surface was adopted for this test. The ambient temperature was 25 ± 2 °C The final value of this test was an average of 2 experimental runs.

#### 2.3.6. Tire Road Testing

Carefully selected tires were mounted onto the factory testing motorcycles according to the internal testing protocol of the corporation. After a mileage of 28,000 km, tire cords were extracted from the tires. The mechanical properties were measured in an Instron tensile tester (gauge length of 190 mm, moving speed of 254 mm/min). The final value of this test was an average of 10 test runs.

#### 2.3.7. Rubber Coverage

The rubber coverage morphology of the cords extracted from the road test tires was evaluated by using scanning electron microscopy (SEM, TESCAN Mira LMS, Brno, Czech), under a high vacuum and 1.5 kV voltage.

A digital microscope (KEYENCE Corporation, Shanghai, China, VHX-7000) was also deployed to analyze the rubber coverage morphology of the tire cords extracted from tires.

## 3. Results and Discussion

### 3.1. Preparation Process for the Nylon-like Polyester Tire Cord

**Figure 2:**
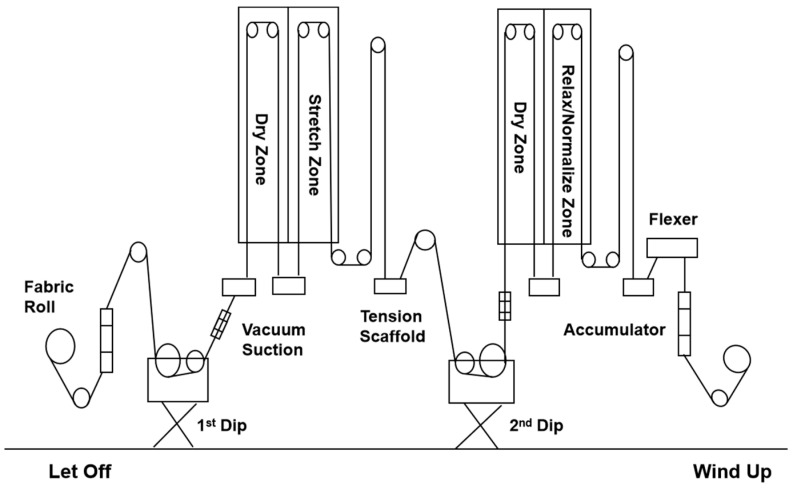
Schematic draft of the dual dipping process (from left to right).

The properties of tire cord are determined by its inner structure. The dipping process parameters have a great impact on the molecular aggregation structure of tire cord because of the temperature and stress field during manufacture [11]. By designing the dipping parameters, a novel nylon-like polyester tire cord was then manufactured to demonstrate the combined characteristics of nylon and polyester tire cords. The whole dipping process is depicted in Figure 2. The polyester greige cord was initially infused into the first dip to introduce polar chemical groups in order to activate polyester’s surface. Before the greige cord was rolled into the Dry Zone, vacuum suctioning was adopted to suck out an extra dip for an energy saving. In the Stretch Zone, polyester tire cord experienced a high temperate to active a chemical reaction of the first dip. The pre-dipped cord was then treated with a second resorcinol formaldehyde latex (RFL) dip to form a three-dimensional network in the Relax Zone. At an elevated temperature, the mobility of the molecular chain of polyester tire cord increased, and the structure of the tire cord was rearranged. The dipping process not only created a polyester tire cord with adequate adhesion to the rubber compound but also affected polyester’s mechanical properties [3,11]. The treatment temperature, time (velocity) and tension are well-known as key parameters in manipulating polyester cord’s thermal mechanical properties [3].

Design of experiment (DOE) was widely used to optimize the dipping process parameters [3], based on practical experience and scientific theory. A two-level fractional factorials DOE experiment (2^7−2^, resolution IV) was then designed to determine the dipping process parameters (Table 1). The polyester greige cord comprised two-ply polyester yarn twisted together (2880 dtex), with a twist level of 370 T/m (Z/S).

A Minitab14 response optimizer could automatically simulate the results of the experiment when changing the dipping parameters in a certain range based on DOE inputs. After analyzing the DOE results, a desired polyester was then manufactured under the treatment parameters in Table 2. A high temperature and low tension in the Relax Zone (238 °C, 150 g) were believed to be the key manufacturing parameters to form this nylon-like polyester tire cord.

### 3.2. Mechanical Properties

**Figure 3:**
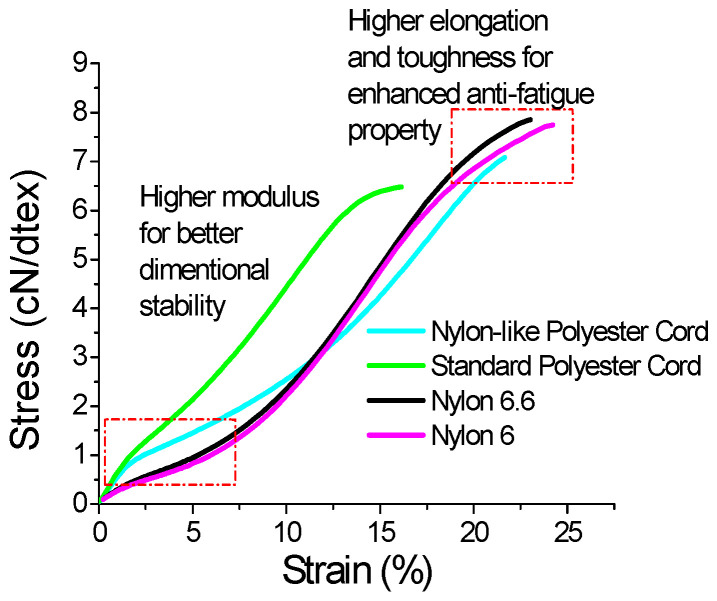
Stress–strain curves of the nylon-like polyester cord and reference cords.

Representative stress–strain curves of nylon-like polyester cord and the reference tire cords are depicted in Figure 3. Compiled data, derived from the stress–strain curves along with thermal shrinkage, are listed in Table 3. In actual service conditions, tire cords are continuously strained at 2–5%. Accordingly, a load at a specified elongation of 5% (LASE-5) was applied to measure the modulus of a tire cord [8,12]. The higher the value of LASE-5, the better the dimension stability it can demonstrate, which is a favorable characteristic of a tire cord. Figure 3 and Table 3 demonstrate that standard polyester tire cords possess higher dimensional and thermal stability, which are the main reasons why the use of polyester tire cords has been increasing in recent years [7]. Nylon cords, both nylon 6 and nylon 6.6, have the highest fatigue resistance, but they have poor dimensional and thermal stability [5]. Nylon-like polyester tire cords have a high modulus typical of standard polyester cords, enhancing the tire dimensional stability and handling performance, as well as a high breaking elongation of up to 21.7%, a favorable characteristic of nylon cords. By integrating the distinctive mechanical properties of nylon and polyester tire cords, nylon-like polyester tire cords assume both practical and required dimensional stability, and good anti-fatigue properties, as well.

### 3.3. Indoor Testing of Tires (Endurance, High Speed, Plunger Energy and Dimensions)

Nylon tire cords have an excellent advantage in impact shock absorbance because of their greater elongation and toughness. Thus, nylon 6 motorcycle tires demonstrate a better performance in rough and poor road conditions. Standard polyester tire cords, on the other hand, have better dimensional stability. This enables passenger car radial tires to have excellent high-speed and handling performance, but the poor impact absorbance performance means these cords are generally not suitable for motorcycle tires [7]. Nylon-like polyester offers an alternative for motorcycle tires. The indoor test results depicted in Table 4 revealed that all nylon-like polyester and nylon 6 experimental tires passed the test standards, but nylon-like polyester tires had slightly lower test values compared to those of the nylon 6 control tires. Nylon cords presented dramatically high thermal shrinkage when subjected to high temperatures. Hence, post-cure inflation (PCI) was required to maintain the shape of the tire during the cooling period. Nylon-like polyester has extremely low thermal shrinkage, allowing the nylon-like polyester tire to retain its contours after being retrieved from a hot tire curing mold [13]. The results showed that PCI elimination for nylon-like polyester tires is becoming possible, which is consistent with the findings of previous studies [5]. PCI elimination could save energy and costs, a positive effect of replacing nylon 6 tire cords with this newly designed nylon-like polyester tire cord.

### 3.4. Tire Dimensional Stability

**Figure 4:**
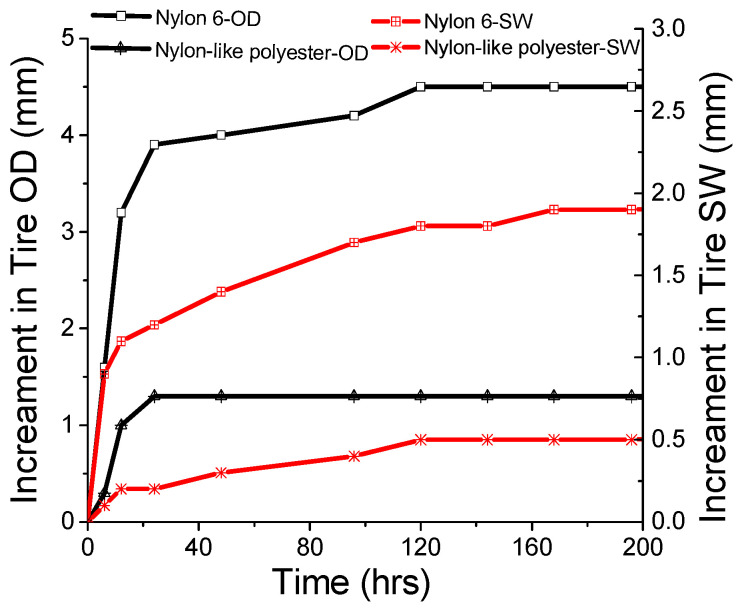
Tire dimensional stability of nylon-like polyester and nylon 6 tire cords over time.

Tire dimensional stability played a role in the performance in terms of uniformity, speed, uneven wear and rolling resistance [14,15]. As indicated in Figure 4, nylon-like polyester tires demonstrated a lower increment in both OD (overall diameter) and SW (section width) compared to those of nylon 6 tires over time, at room temperature, showing a better dimensional stability. The dimensional stability performance of nylon-like polyester tire cord was derived from its higher modulus.

### 3.5. Rolling Resistance

**Figure 5:**
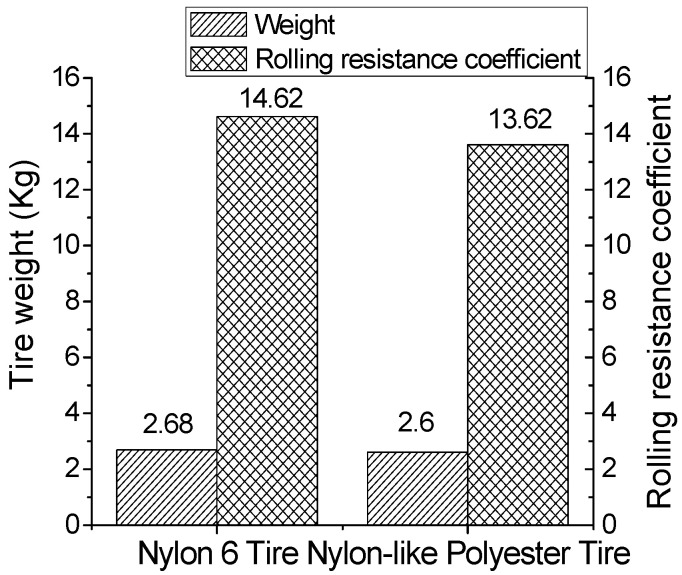
Rolling resistance experiment of nylon-like polyester and nylon 6 tires.

Tire rolling resistance is the force that resists the rolling of a tire along the road surface, which, in turn, means a tire is constantly changing its shape in service. The rolling resistance is essentially a hysteresis loss of viscoelastic materials, such as rubber and synthetic tire cord [16]. The rolling resistance is interpreted as the mechanical energy that is transformed into heat as the tire travels for a unit of distance [14]. In the past few decades, the rolling resistance of tires has been reduced dramatically, partly due to the better understanding of the principles regarding rolling resistance, the development of new, less hysteretic materials and the adoption of new tire structure designs [17]. The tire tread component accounts for a large proportion of the amount of energy loss. Efforts have been made to reduce the hysteresis loss of tread rubber compounds without sacrificing safety and other technical properties [14,16,17]. Tire cords also play a crucial role in reducing rolling resistance, and the proportion may vary considerably, given different types of tires [18]. In this study, as demonstrated in Figure 5, when the tire weight was the same, the rolling resistance coefficient of a nylon-like polyester motorcycle tire was reduced by 6.8% compared to that of nylon 6 cord, which was due to the high modulus of nylon-like polyester tire cord. This can notably help in achieving fuel savings and reducing CO_2_ emissions. A previous study showed that 5–15% of fuel consumption was removed as a result of controlling the rolling resistance in passenger cars, and 15–30% for heavy duty vehicles [14].

### 3.6. Road Testing of Tires

The experimental tires were mounted on a HONDA 18-inch motorcycle vehicle for the evaluation of on-road performance. Feedback (Table 5) from the professional driver (utilized for this study) revealed that their handling and ride comfort performance were better than those of the nylon 6 control tires. The higher modulus of nylon-like polyester tire cord was the reason for the better handling performance. After the field test, neither the nylon-like polyester tires nor the nylon 6 control tires had visible damage.

**Figure 6:**
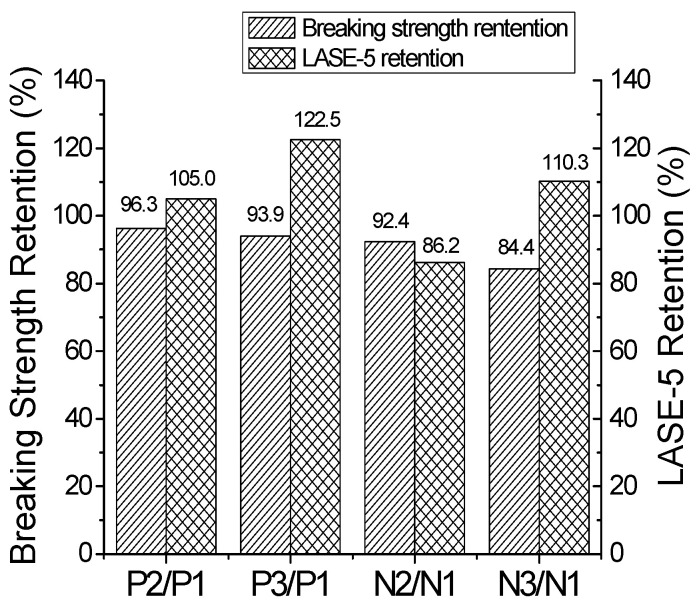
Retention of breaking strength and LASE-5 of cords extracted from road test tires.

The mechanical properties of tire cords extracted from new tires and the field test tires are given in Table 6 and Figure 6. The working length in the Instron tensile tester was 190 mm. The breaking strength retention of nylon-like polyester tire cords we extracted was maintained at a higher level than that of nylon 6 tire cords in both the new tires and field test tires. There were no major differences in the retention of breaking elongation. As mentioned earlier, LASE-5 is an indicator of the tire cord modulus. Nylon-like polyester cords had a higher retention than that of nylon 6 cords extracted from the tires after a road test. A higher modulus in nylon-like polyester cords could result in better handling and greater ride comfort performance of the tire [7].

**Figure 7:**
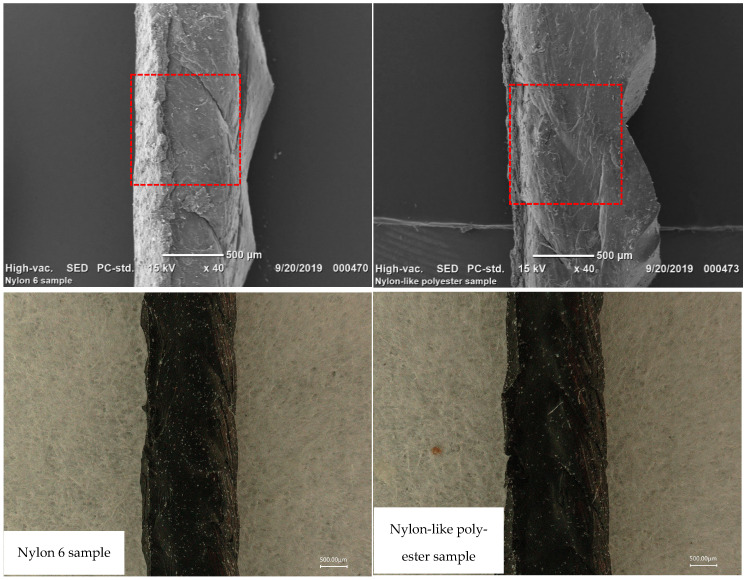
Rubber coverage of nylon-like polyester and nylon 6 cords extracted from road test tires.

The results were consistent with the feedback from road testing. The extracted tire cords’ surfaces were evaluated via SEM and a digital microscope. Figure 7 reveals that both nylon-like and nylon 6 extracted cords had a good rubber coverage, indicating that adhesion failure occurred in the rubber compound matrix [19,20]. Nylon-like polyester tire cord had comparable adhesion properties to nylon 6 tire cord after the 28,000 km road test. In our previous study, nylon-like polyester tire cord exhibited a similar normal curing adhesion performance to that of nylon 6, which was consistent with road test adhesion results [3].

## 4. Conclusions

A novel nylon-like polyester, which combines the characteristics of nylon and polyester tire cords, was designed by optimizing the dipping process parameters. The performance of a nylon-like polyester motorcycle tire was then evaluated. The results showed that the nylon-like polyester motorcycle tire passed all the indoor tests. We also found that the elimination of PCI was possible, resulting in energy and cost savings. The rolling resistance coefficient was reduced by 6.8% compared to that of the nylon 6 control tire. The handling and ride comfort performance were improved, as well. Rubber coverage morphological pictures showed that the nylon-like polyester cord had comparable adhesion to the nylon 6 cord after a road test. Based on our thorough validation and analysis, we conclude that nylon-like polyester tire cords can enhance the performance of motorcycle tires and can eventually result in energy savings, when replacing nylon 6 tire cord with nylon-like polyester tire cord.

## Figures and Tables

**Table 1:** Design of experiment (DOE) of the dipping thermal treatment parameters.

	Temperature Setting of Zone (°C)			Tension Setting of Zone (g)			Velocity (m/min)
	Dry	Stretch	Relax	Dry	Stretch	Relax	
Level 1	150	180	180	700	600	150	6
Level 2	170	240	246	1100	1200	600	18

**Table 2:** Result of design of experiment (DOE) of dipping parameters.

	Temperature Setting of Zone (°C)			Tension Setting of Zone (g)			Velocity (m/min)
	Dry	Stretch	Relax	Dry	Stretch	Relax	
Result	160	180	238	1000	900	150	15

**Table 3:** Compiled mechanical properties of the nylon-like polyester cord and reference cords.

Item	Unit	Nylon-like Polyester Cord	Standard Polyester Cord	Nylon 6	Nylon 66
Nominal linear density	dtex	2880	2880	2800	2800
Toughness	J	5.1	4.3	5.9	6.3
Breaking tenacity	cN/dtex	7.08	6.47	7.74	7.85
Breaking elongation	%	21.7	16.2	24.2	23.1
Diameter	mm	0.65	0.62	0.64	0.64
LASE-5	N	41.9	61.6	23.0	26.3
Thermal shrinkage	%	0.1	1.2	6.9	4.9

**Table 4:** Indoor testing results of tires with nylon-like polyester and nylon 6 tire cord fabrics.

Indoor test	Tire nominal specification	3.00–18 motorcycle bias tire		
	Fabric specification	Nylon-like polyester 1440 dtex/2 23 EPI		Nylon 6 1400 dtex/2 20 EPI
Endurance	Test standard IS 15627:2005 [9]	>34 h (internal target 70)		
	Accumulated time (h)	75		90
	Accumulated distance (km)	4500		5400
	Conclusion	Pass		Pass
Plunger energy	Test standard IS 15627:2005 [9] (plunger diameter: 8.0 mm)	>45 J		
	Actual value (J)	68.5		74
	Conclusion	Pass		Pass
High speed	Test standard IS 15627:2005 [9]	>140 Km/h, 60 min		
	Accumulated time (min)	116		119
	Accumulated speed (Km/h)	200		200
	Conclusion	Pass		Pass
Dimension after inflation	Test standard of overall diameter	IS 15627 2005 [9]: 623–639 (mm)		
	Actual value (mm)	PCI	PCI eliminated	PCI
		638.2	631.5	638.2
	Conclusion	Pass		Pass
	Test standard of section width	IS 15627 2005: 77–86 (mm)		
	Actual value (mm)	PCI	PCI eliminated	PCI
		83	80	83
	Conclusion	Pass		Pass

**Table 5:** Ride and handling performance of nylon-like and nylon 6 motorcycle tires.

Ride and Handling Test	Nylon-like Tire	Nylon 6 Tire
Handling shimmy	7.3	7.0
Straight stability	8.0	7.0
Large curve testing	8.0	7.0
Slalom	8.0	7.0
Braking stability	7.0	7.0
Ride comfort	7.5	7.0
Wobbling	7.0	7.0

**Table 6:** Mechanical properties of nylon-like polyester and nylon 6 cords extracted.

Code	Description	Breaking Strength (N)	Breaking Elongation (%)	LASE-5 (N)
P1	Nylon-like polyester dipped cord	203.3	23.54	40
P2	Nylon-like polyester cord extracted from a new tire	195.7	21.82	42
P3	Nylon-like polyester cord extracted from a field test tire—28,000 Km	190.8	20.23	49
N1	Nylon 6 dipped tire cord	225.3	23.23	29
N2	Nylon 6 tire cord extracted from a new tire	208.1	21.62	25
N3	Nylon 6 tire cord extracted from a field test tire—28,000 Km	190.1	18.88	32

## Data Availability

For the detailed preparation data of this nylon-like polyester tire cord, please refer to https://journals.sagepub.com/doi/10.1177/1558925018825271, accessed on 10 February 2019.
